# Impact of land configuration and organic nutrient management on productivity, quality and soil properties under baby corn in Eastern Himalayas

**DOI:** 10.1038/s41598-020-73072-6

**Published:** 2020-09-30

**Authors:** Subhash Babu, Raghavendra Singh, R. K. Avasthe, Gulab Singh Yadav, Anup Das, Vinod K. Singh, K. P. Mohapatra, S. S. Rathore, Puran Chandra, Amit Kumar

**Affiliations:** 1 ICAR Research Complex for North Eastern Hill Region, Umiam, Meghalaya 793 103 India; 2 ICAR-National Organic Farming Research Institute, Gangtok, Sikkim 737 102 India; 3 ICAR-Research Complex for North Eastern Hill Region, Tripura Centre, Lembucherra, West Tripura, 799 210 India; 4 Division of Agronomy, ICAR-Indian Agricultural Research Institute, New Delhi, 110 012 India; 5 ICAR-National Bureau of Plant Genetic Resources, New Delhi, 110 012 India

**Keywords:** Plant sciences, Environmental sciences

## Abstract

Appropriate land configuration and assured nutrient supply are prerequisites for quality organic baby corn (*Zea mays* L.) production in high rainfall areas of the delicate Eastern Himalayan Region of India. A long term (5-year) study was conducted during 2012–2016 on a sandy loam soil in the mid attitude of Sikkim, Eastern Himalayan Region of India to evaluate the productivity, produce quality, the profitability of baby corn, and soil properties under different land configurations comprising flatbed, ridge and furrow, and broad bed and furrow, and organic nutrient management practices comprising un-amended control, farmyard manure 12 t ha^−1^, vermicompost 4 t ha^−1^ and farmyard manure 6 t ha^−1^ + vermicompost 2 t ha^−1^. The baby corn sown on broad bed and furrow had the tallest plant (149.25 cm), maximum dry matter (64.33 g plant^−1^), highest leaf area index (3.5), maximum cob length (8.10 cm), cob girth (6.13 cm) and cob weight (8.14 g) leading to significantly higher fresh baby corn yield (1.89 t ha^−1^), and net returns (US$ 906.1 ha^−1^) than those of other treatments. Mineral composition (phosphorus, potassium, iron, and zinc), protein, and ascorbic acid content were also the highest in baby corn grown under the broad bed and furrow system. The soil of broad bed and furrow had a higher pH, organic carbon content, organic carbon pools, microbial biomass carbon, and enzymatic activities (dehydrogenase, fluorescein diacetate, and acid phosphatase) compared to soils of other land configurations. A combined application of farmyard manure (6 t ha^−1^) + vermicompost (2 t ha^−1^) improved the crop growth and produced 117.8% higher fresh baby corn and 99.7% higher fodder yield over control (0.9 t fresh corn and 13.02 t fodder yield ha^−1^), respectively. This treatment also registered significantly higher gross return (US$ 1746.9 ha^−1^), net return (US$ 935.8 ha^−1^), and benefit–cost ratio (2.15) than other nutrient management practices. Fresh cob quality in terms of protein (22.91%) and ascorbic acid content (101.6 mg 100 g^−1^) was observed to be significantly superior under combined application of farmyard manure (6 t ha^−1^) + vermicompost (2 t ha^−1^) than those of other nutrient management systems. However, fresh baby corn cobs produced with vermicompost 4 t ha^−1^ had the highest concentration of phosphorus, potassium, iron, and zinc. Application of farmyard manure 12 t ha^−1^ registered the maximum increment in soil organic carbon content (1.52%), its pool (40.6 t ha^−1^) and carbon sequestration rate (0.74 t ha^−1^ year^−1^) followed by integrated application of farmyard manure (6 t ha^−1^) and vermicompost (2 t ha^−1^). The maximum soil microbial biomass carbon and enzymatic activities [dehydrogenase (22.1 µg TPF g^−1^ soil h^−1^) and fluorescein diacetate (67.1 µg FDA g^−1^ soil h^−1^)] were noted with the combined use of farmyard manure (6 t ha^−1^) + vermicompost (2 t ha^−1^). Thus, the study suggests that the broad bed and furrow land configuration along with the combined application of farmyard manure + vermicompost could be an economically feasible practice for quality organic baby corn production and soil health improvement in the Eastern Himalaya and other similar eco-regions elsewhere.

## Introduction

Globally, public awareness about the hazardous effects of irrational use of agrochemicals on human health increased the demand for safe and organically grown food^1^. With the growing anxiety over health, people prefer quality nutritious food than bulky stuff. With increasing consumer preferences and purchasing power, people are ready to buy organic food products at a much higher price than conventional products^2,3^. It is established that organic farming products are superior in quality, safe, and environment-friendly than the produce from synthetic chemical-based farming^3,4^. In addition to this, organic farming has the minimum negative impact on environmental quality^5,6^, human and animal health^7^, and it improves the soil structure^8^, soil organic carbon (SOC)^9^, soil functionality^10^, water use efficiency^11^ and ecosystem services^12,13^. However, some researchers indicated a 20–40% yield reduction in organic farming compared to that of chemical-based farming^14,15^. The available data indicates that the magnitudes of yield reduction depend on the types of crops grown, the climate, soil conditions, and management practices^7^. Consequently, organic farming systems may require additional land to produce the same quantity of output to that of conventional farming^11,15^. This warrants the cultivation of short duration, high value, high volume crops like vegetables to compensate for the yield losses by fetching a premium price (a price higher than the standard price for products/goods). Organic produce especially vegetables are important for nutritional security^16^ and for obtaining premium price^17^. In the case of most of the vegetable crops, the entire biomass is removed from the field as economic output, which accelerates soil carbon losses^18^. Furthermore, the nutrient’s requirement of vegetable crops is relatively higher as compared with other crops like cereal and legumes^3^. Hence, nutrient management is a major issue in organic vegetable production systems^19^. One such potential vegetable for organic production is baby corn (*Zea mays* L.). Baby corn is a cereal crop that is harvested at the onset of silking and can be consumed as a vegetable while the stalks are still immature^20^.

Baby corn is a high-value short duration (65–75 days) multi-use cereal crop, well-suited to intensive cropping systems, and can be grown throughout the year under diverse climatic conditions. Baby corn production and markets are growing worldwide, especially in Asia, Africa, and South America^21^. Asian countries are the major consumers of baby corn. In Asia, Thailand, China, and Taiwan are the major baby corn producers^21^. Similar to other Asian countries, in India, it is gaining attention among the growers owing to its high demand, promising market, value addition, and high-income opportunities. Baby corn is a rich source of crude protein, phosphorus (P), potassium (K), calcium (Ca), sugars, ascorbic acid, and crude fiber content^21^. Baby corn is used either of two ways, fresh or processed consumption^22,23^. Besides the main product, it also provides a considerable amount of quality green fodder; a by-product, a valuable feed for cattle^24^. Hence, the cultivation of baby corn provides an opportunity to maintain a dairy farm. Livestock is an integral component of organic farming as the sustainability of a low input production system depends on close nutrient recycling of on-farm inputs. Nutrient management in organic production is a major challenge for obtaining profitable yield^25^. Therefore, nutrients’ supply is more crucial in high-density short duration crops like baby corn^20^ when it is grown under organic farming. Adequate and consistent supply of nutrients during the entire growth period (seedling to harvesting) of baby corn is indispensable for harvesting optimum economic yield^26^. Under organic management, nutrient’s release and crop demand synchrony is very much required^27^; hence, a thorough understanding of nutrient’s release pattern from organic sources is essential to avoid nutrients stress. Thus, the development and implementation of efficient nutrient management practices are pivotal for successful organic baby corn production^28^ and to improve the product quality and yield, besides overall soil health improvement^2^.

Baby corn is highly sensitive to both excess and deficit moisture stress. Water stagnation arrests/minimizes most of the soil enzymatic activities leading to the poor transformation of soil nutrients. Hence, adequate drainage facilities are pre-requisites for successful baby corn production in high rainfall areas. Land configuration i.e., alteration of land surface and shape of seedbed will provide an opportunity to facilitate the drainage of excess water and avoid the water stagnation on the soil surface. Land management system plays a crucial role in minimizing soil erosion and improving the water use efficiency of field crops^29^. However, most of the research on a land configuration has focused on avoiding/minimizing drought stress in crops especially in semi-arid, and tropical and subtropical climates^30^ especially in mustard (*Brassica juncea*)^31^, pearl millets (*Pennisetum glaucum*)^32^, and corn^33^. However, systematic information on the effect of land configuration on baby corn productivity and product quality for high rainfall high altitude areas is not available to policymakers and farmers.

The Eastern Himalayas outspread from the Kaligandaki Valley in Nepal to northwest Yunnan in China—encompassing Bhutan, the eight Indian states, part of Tibet, Myanmar and spreads over ~ 52.5 million hectares (Mha)^34^. The North Eastern Himalayan Region (NEHR) of India is a high rainfall zone covering > 26.3 Mha area^35^ and receives ~ 250 cm rain per annum, which has a very high potential for organic baby corn production because of its unique climatic conditions for crop production. However, heavy rains during pre-monsoon and monsoon season cause water stagnation, thereby, adversely affecting the growth and yield of crops. Inadequate drainage is the main constraint for profitable baby corn production in the NEHR and other areas with similar climatic conditions. However, with the development of a suitable land configuration system for crop establishment, three crops of baby corn can be grown in a year in high rainfall area of the hilly region by facilitating adequate drainage. Many food processing companies are evolving in the NEHR. There is tremendous scope for contract organic farming of baby corn for assured income to the growers in the region. Furthermore, baby corn has higher employment generation potential as compared with traditional crops of the region under organic management^36^. However, baby corn is a nutrient demanding crop, requires heavy fertilization especially under high rainfall zones like NEHR. Agriculture in the NEHR, India is organic by default and the Hon’ble Prime minister of India declared Sikkim as the first organic farming state of India in January 2016. The National Programme for Organic Production (NPOP), Government of India legislation was applied to declare Sikkim as the first organic state in India. Other states of the NEHR also intend to become organic states in the near future. However, productivity under organic farming largely depends on an assured supply of nutrients through organic manures. Basal application of farmyard manure (FYM) at the time of land preparation is a common practice in organic farming. The basal application of FYM is not sufficient to support the nutrient demand of crops from seeding to harvesting due to its low nutrient content and very slow release pattern. This makes the synchronization of nutrients release and their uptake by crop plants very difficult, especially in short duration crops like baby corn. Many studies have demonstrated that the combined application of organic manures enhances the baby corn yield over their sole applications in the semi-arid region of India^37^, the Western Himalayan region^38^, the Indo-Gangetic Plains of India^39^ and the Eastern Gangetic Plains^33,40^. However, these recommendations are not directly replicable in high rainfall zones of the NEHR, India due to variations in pedo-climatic, socio-economic, and technological constraints. Studies have not been conducted in the NEHR to evaluate the impact of land configuration and integrated use of organic manures on baby corn productivity, profitability, quality, and soil health.

Thus, it was hypothesized that broad bed and furrow (BBF) sowing and conjoint use of FYM and vermicompost (VC) may be an economically viable practice for quality organic baby corn production, besides sustaining the soil health in the NEHR and similar hill eco-regions across the globe. The specific objectives of the experiment to test the above hypothesis were to i) ascertain the effect of land configuration and organic nutrient management practices on productivity, quality, and economics of baby corn in high rainfall zone; and ii) evaluate the changes in soil carbon, available nitrogen (N), phosphorus (P), potassium (K) and enzymatic reactions in response to land configuration and organic nutrient management practices.

## Materials and methods

### Experimental site

**Figure 1:**
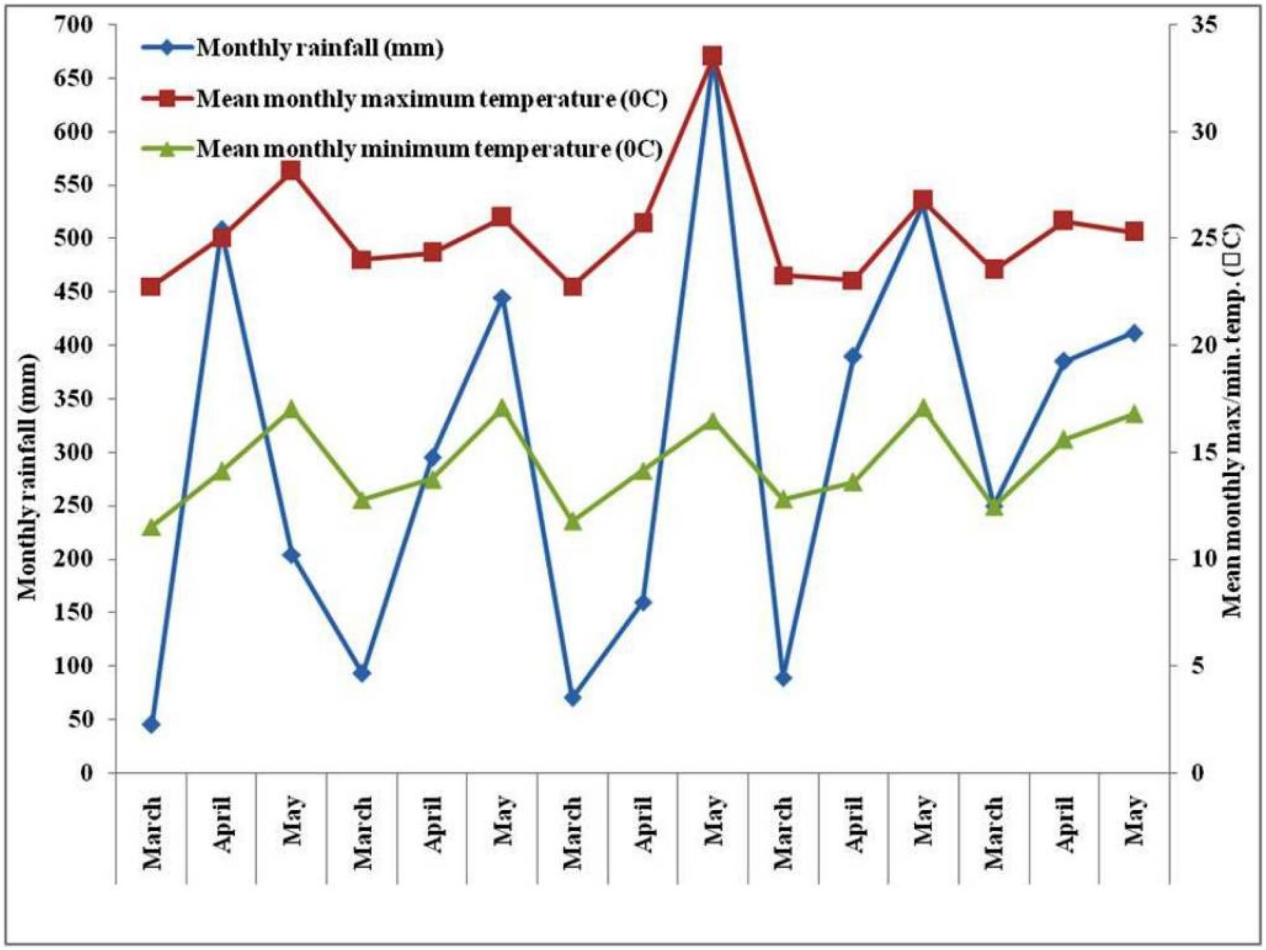
Monthly rainfall (mm), mean monthly maximum and minimum temperatures (°C) during experimental period.

Field investigations were conducted during five consecutive pre-*Kharif* seasons (2012–16) at the Research Farm, Indian Council of Agricultural Research (ICAR) Research Complex, Sikkim Centre, Tadong, Gangtok, Sikkim, India. The experimental site is located at 27^o^32′ N latitude and 88^o^60′ E longitude at an altitude of 1350 m above mean sea level. The experimental site had a mild temperate climate. The mean annual maximum and minimum temperatures were 22 °C and 4 °C, respectively. The experimental field was under organic management since 2003. The *Haplumbrept* soil of the experimental site was sandy loam in texture. Composite pre-experiment soil samples were taken from 0–20 cm depth for initial soil analysis. The pre-experiment values of different soil parameters (0–20 cm) are presented in Table 1. Meteorological observations during the experimental period are presented in Fig. 1.

**Table 1:** Soil properties (0–20 cm) at the initiation of the experiment (2012).

Parameters	Values	References
Sand (%)	44.6	Piper^90^
Silt (%)	41.6	Piper^90^
Clay (%)	13.8	Piper^90^
Organic carbon (%)	1.34	Walkley and Black^49^
Bulk density (Mg m^−3^)	1.37	Blake and Hartge^44^
pH (1:2 soil: water)	6.15	Prasad et al.^41^
Available–N (kg ha^−1^)	336.4	Prasad et al.^41^
Available–P (kg ha^−1^)	14.8	Prasad et al.^41^
Available–K (kg ha^−1^)	352.8	Prasad et al.^41^
Microbial biomass carbon (µg MBC g^−1^ soil)	318.3	Vance et al.^45^
Fluorescin di acetate (µg FDA g^−1^ soil h^−1^)	38.61	Green et al.^47^
Dehydrogenase activity (µg TPFg^−1^ soil h^−1^)	11.51	Casida et al.^46^
Acid phosphatase (µg p-nitrophenol g^−1^ soil h^−1^)	2.14	Tabatabai and Bremner^48^

*N* nitrogen, *P* phosphorus, *K* potassium, *MBC* microbial biomass carbon, *TPF* triphenylformazan, *FDA* fluorescin di acetate.

### Experimental setup and crop management

**Figure 2:**
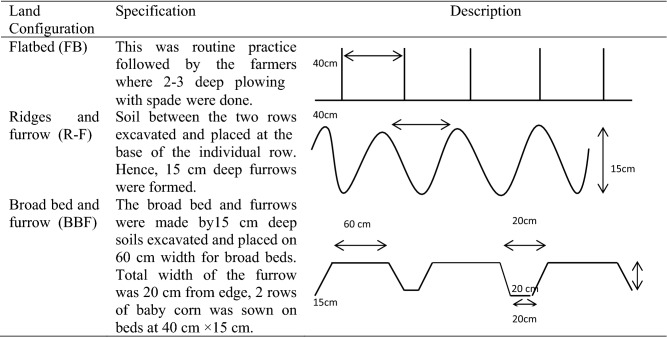
Description of land preparation.

A set of 12 treatment combinations comprising of three land configurations viz*.,* flatbed sowing (FB, it is common farmers’ practice), flat sowing followed by earthing up after 20 days of sowing (ridge and furrow-RF) and sowing on broad bed and furrow (BBF) were assigned to main plots and four organic nutrient management practices viz*.,* non-amended (control), FYM 12 t ha^−1^, VC 4 t ha^−1^ and FYM 6 t ha^−1^ + VC 2 t ha^−1^ allocated to sub-plots and tested under split-plot design with three replications for five consecutive years on the same field (treatments superimposed). Well decomposed organic manure (FYM and VC) was applied manually in each plot, 1 week before sowing, and mixed well into the top-15 cm soil. The nutrient composition of FYM and VC is presented in Table 2. To avoid the intermixing of the different manures between the subplots, each subplot and main plot was separated by a permanent bund (75 cm width and 10–15 cm height), which were repaired and maintained every year during the entire period of the study. The individual plots were manually prepared/plowed with the help of spade during each cropping season. Baby corn (cultivar G-5406) was sown manually with a spacing of 40 cm × 15 cm during the first week of March every year. Land configurations were made each year. On the FB, the sowing of baby corn was done by maintaining the normal distance (40 cm × 15 cm). However, in the RF method, 15 cm deep soil was excavated 20 days after sowing (DAS) between two rows of baby corn and placed at the base of individual rows. Under the BBF system, the width of the bed and furrow was 60 cm and 20 cm, respectively, and two rows of baby corn were planted on the bed at a spacing of 40 cm. A detailed description of land preperation/configurations along with the schematic diagram is presented in Fig. 2. After sowing, the seeds were covered with soil and gap-filling/thinning was undertaken 1 week after sowing to maintain a uniform plant population. No major incidence of insects-pests and diseases were observed during the crop growth period, hence, no plant protection measures were required. However, irrespective of the treatment one hand weeding was given at 20 DAS. The crop was de-tasseled (removal of male inflorescences) before initiation of pollen shedding (55–60 DAS) to prevent fertilization and divert nutrient flow towards the baby cobs.

**Table 2:** Nutritional composition of organic manures used in experimentation (2012–2016).

Particulars	Farmyard manure	Vermicompost
pH	6.48 ± 0.29	6.94 ± 0.09
EC (ds m)	0.14 ± 0.02	0.16 ± 0.04
N (%)	0.61 ± 0.09	1.56 ± 0.05
P (%	0.29 ± 0.04	0.85 ± 0.05
K (%)	0.52 ± 0.05	1.25 ± 0.10
Zn (ppm)	72.8 ± 11.4	384.2 ± 21.24
Fe (ppm)	1674 ± 299.6	5606.6 ± 1082.2
Mn (ppm)	42.8 ± 10.8	341.0 ± 29.54
Cu (ppm)	189.6 ± 32.8	237.20 ± 25.64

*EC* electrical conductivity, *N* nitrogen, *P* phosphorus, *K* potassium, *Zn* zinc, *Fe* iron, *Mn* manganese, *Cu* copper.

### Harvesting, growth and yield measurement

Fresh baby cobs along with attached sheaths were harvested at 2–3 days interval in between 65–75 days after sowing (within 2–3 days of silking) during the first week of May every year. At the time of harvesting, five plants were randomly selected from each plot for the recording of growth parameters. Plant height was measured from base to the tip of a leaf and expressed in cm. Leaf area index (LAI) i.e., the leaf area per unit land area was calculated. Five plants were uprooted from the sampling row, cleaned and separated from the root portion, oven-dried at 70 ± 5 °C till achieving constant dry weight, and expressed in g plant^−1^ for estimation of dry matter accumulation (DMA). After harvesting randomly 10 cobs from each harvest were selected from each plot. The cob sheath of selected baby cobs was peeled-off, cob length, and weight were recorded and averaged from which mean values were attained. The cob girth was measured by vernier calipers and the mean value was presented. Baby corns were de-husked manually and the yields of baby corns were reported on a fresh weight basis and expressed in t ha^−1^. After complete harvesting of baby cobs from the plants, the entire biomass was removed plot-wise with the help of iron sickle from the field, weighed and expressed as t ha^−1^.

### Quality analysis

Baby corn samples were collected at harvest, oven-dried at 70 ± 5 °C, and ground. To remove the foreign particles, the samples were passed through a 40-mesh sieve. The grounded material was collected in butter paper bags for chemical analysis. The total nitrogen content in cob samples was analyzed by nitrogen determination in KJELTEe, AUTO 1030. For the estimation of P, 10 ml of diacidic (nitric acid: perchloric acid 3:1) mixture was poured into a 150 ml conical flask containing one gram of finely pulverized baby corn. The mixture was swirled to mix the baby corn thoroughly with the diacid. The flask was placed on a hot plate until the digestion was completed. After the digestion, the aliquot was passed through a Whatman grade 40 filter paper and the filtrate was collected in a 100 ml volumetric flask. The final volume was made up with distilled water. 10 ml of the aliquot was taken and mixed with 10 ml ammonium molybdate solution and mixed. P content was recorded in a spectrophotometer (Model GENESYS 10 UV)^41^. Similarly, for the estimation of total K content, one gram finely ground baby cob was mixed with 20 ml of the acid mixture (conc. HNO_3_: conc. H_2_SO_4_: HClO_4_) (5:1:2) in a 100 ml conical flask. The flask was placed on a hot plate until the digestion was completed. The flask was removed from the hot plate and allowed to cool. After cooling, 20 ml of distilled water was added and the aliquot was filtered through Whatman filter paper grade 40 into a 100 ml volumetric flask. The final volume adjusted and the aliquot was used for estimation of K by a flame photometer (SYSTRONIC)^41^. Micronutrients [Iron (Fe) and Zinc (Zn)] content were estimated by Atomic Absorption Spectrophotometer (Model GBC 932 plus). The protein content in the cob was obtained by multiplying the total N content with constant factor 6.25 and expressed in percentage. The ascorbic acid content in fresh baby corn was estimated by the 2, 6 dichlorophenol indophenol dye method^42^ and expressed in mg 100 g^−1^.

### Manure sampling and analysis

Every year, manure (FYM and VC) samples were collected immediately after application from four places in each plot. The collected samples from different plots were mixed to form a composite sample each for FYM and VC. Manure samples were collected in a transparent plastic bottle, sealed tightly and stored in a refrigerator (4 °C) until individual parameters were analyzed. The samples were air-dried, ground, passed through a 2 mm sieve for analysis of pH and EC. The total N, P, and K were analyzed by the Kjeldahl, ammonium vanadomolybdate absorptiometric analysis, and flame photometry methods, respectively^43^. The micronutrients (Fe, Mn, Zn, and Cu) content were determined by using an atomic absorption spectrophotometer^43^.

### Soil sampling and processing

After completion of five cropping cycles, soil samples were collected using 10 cm scaled-soil cores with 5.4 cm inner diameter from 0 to 20 soil depth from each plot. Soil samples were collected randomly from four places in each plot and then blended for a representative composite soil sample. Soil bulk density (ρ_b_) was determined by the core method^44^ after oven drying at 105 ± 1 °C. The collected bulk soil samples were air-dried at room temperature (25 °C); the clods were broken manually and sieved with 2 mm sieve. The processed soil samples were stored in airtight plastic bags for analysis of SOC, pH, available N, P, and K. Part of representative fresh soil samples from each plot and were stored at freezing temperatures for analyzing the MBC, DHA, FDA, and acid phosphatase activities.

### Soil biochemical analysis

The soil MBC was determined by the chloroform fumigation-extraction method^45^ and expressed in µg MBC g^−1^ soil. The DHA was estimated by reducing 2, 3, 5-triphenyl tetrazolium chloride (TTC)^46^ and expressed in terms of mg formazan g^−1^ dry soil hr^−1^. The FDA was estimated as per the method suggested by Green et al.^47^. Acid phosphatase activity was estimated^48^ and expressed as a mole of p-nitrophenol released g^−1^ dry soil h^−1^. Available-N (Alkaline KMnO_4_ method), available-P (Bray’s P_1_, 0.03 N NH_4_F in 0.025 N HCl, pH 4.65), available-K (1 N NH_4_OAc extractable K, pH 7.0) and soil pH (soil and water ratio 1:2.5) were estimated by the procedure outlined by Prasad et al.^41^.

### Computation of soil carbon pool and estimation of carbon sequestration

The concentration of SOC was analyzed by the wet oxidation method^49^. The SOC value was assumed to be equal to total soil C with negligible inorganic C concentration as the pH of the soil was < 7.0^50^. The SOC pool (Mg ha^−1^) at 0–20 cm depth was calculated by using the following equation:

$${\text{SOC}}\;{\text{or}}\;{\text{C}}\;{\text{fraction}}\;{\text{pool}}\;{\text{(t}}\;{\text{ha}}^{ - 1} {)} = \frac{{{\text{ SOC}}\;{\text{Concentration}}\;\% }}{100} \times \frac{{{\text{Depth}}\;{\text{(cm) }}}}{100} \times \frac{\rho b Mg}{{{\text{m}}^{3} }} \times \frac{{Area (10,000\;{\text{m}}^{2} )}}{{{\text{ha}}}}$$

The accumulation of SOC was computed with the following equation:

$${\text{SOC}}\;{\text{accumulation}}\;{\text{(t}}\;{\text{ha}}^{ - 1} {)} = {\text{Final}}\;{\text{SOC }}\;{\text{pool }}\;{\text{(t}}\;{\text{ha}}^{ - 1} {)} - {\text{Initial}}\;{\text{SOC }}\;{\text{pool}}\;{\text{(t}}\;{\text{ha}}^{ - 1} {)}$$

The sequestration of the SOC was computed as per the following equation:

$${\text{SOC}}\;{\text{sequestration}}\;{\text{(t}}\;{\text{ha}}^{ - 1} \;{\text{year}}^{ - 1} {)} = \frac{{{\text{ Final}}\;{\text{SOC }}\;{\text{pool}}\;{\text{ (t}}\;{\text{ha}}^{ - 1} {)} - {\text{Initial}}\;{\text{SOC}}\;{\text{ pool}}\;{\text{(t}}\;{\text{ha}}^{ - 1} {)}}}{Period\; of\; the\; study\; (Years)}$$

### Economic analysis

The benefit–cost analysis was performed to test the economic feasibility of different land configurations and organic nutrient management practices for baby corn production. The cost of baby corn production was calculated based on different inputs used and crop management practices followed like tilling, seed, manuring, labor employed, sowing, intercultural operations, weed management, and harvesting. The gross return (GR) was the market value of the main (baby corn) and by-products (green fodder). The net return (NR) and benefit to cost ratio (B:C ratio) were calculated by the following expressions.

$$\begin{aligned} & {\text{NR}}\;{\text{(US}}\$ \, \;{\text{ha}}^{{ - {1}}} {) } = {\text{ Gross}}\;{\text{ return }}\;{\text{(US}}\$ \;{\text{ ha}}^{{ - {1}}} {) }{-}{\text{ Cost}}\;{\text{ of}}\;{\text{ cultivation}}\;{\text{ (US}}\$ \;{\text{ ha}}^{{ - {1}}} {)} \\ & {\text{B}}:{\text{C}}\;{\text{ratio }} = {\text{ Gross}}\;{\text{ return}}\;{\text{ (US}}\$ \, \;{\text{ha}}^{{ - {1}}} {)}/{\text{Cost }}\;{\text{of }}\;{\text{cultivation}}\;{\text{ (US}}\$ \, \;{\text{ha}}^{{ - {1}}} {)} \\ \end{aligned}$$

### Statistical analysis

The general linear model (SAS Institute, Cary, NC) procedure was used to compute the ANOVA for split-plot design to determine the statistical significance between the various land configurations and organic nutrient management practices. A comparison between treatment means was done as per the procedure of Gomez and Gomez^51^.

## Results

### Growth and yield attributing parameters of baby corn

Land configurations had a significant effect on growth (plant height, LAI, and dry matter accumulation plant^−1^ at harvest) and on yield attributing parameters (cob length, girth, and weight of individual cob) of baby corn (Table 3). Among the land configurations, baby corn on BBF produced the tallest plant (149.25 cm), having the maximum LAI (3.5), DMA (64.33 g plant^−1^), cob length (8.10 cm), girth (6.13 cm) and weight (8.14 g). Plants under BBF had 20.3, 12.5, 30.8, and 38.2% higher plant height, LAI, DMA, and cob weight than that under FB, respectively.

**Table 3:** Effect of land configuration and organic nutrient management practices on growth and yield contributing characteristics of baby corn (Five years avg.).

Treatments	Plant height (cm)	Leaf area index	DMA (g plant^−1^)	Baby corn length (cm)	Baby corn girth (cm)	Baby corn weight (g)
**Land configuration**						
FB	124.08	3.11	49.17	5.78	4.08	5.89
RF	137.33	3.19	59.00	6.98	5.09	7.16
BBF	149.25	3.50	64.33	8.10	6.13	8.14
SEm ±	0.62	0.013	0.37	0.05	0.05	0.05
LSD (*p* = 0.05)	1.83	0.038	1.10	0.16	0.15	0.14
**Organic nutrient management practices**						
Un-amended control	127.44	3.14	50.11	6.31	4.50	6.45
FYM 12 t ha^−1^	132.67	3.24	54.22	6.63	4.95	6.78
VC 4 t ha^−1^	139.78	3.29	59.22	7.13	5.34	7.40
FYM 6 t ha^−1^ + VC 2 t ha^−1^	147.67	3.39	66.44	7.72	5.61	7.64
SEm ±	0.72	0.015	0.43	0.06	0.06	0.05
LSD (*p* = 0.05)	2.11	0.044	1.27	0.18	0.17	0.16

*FB* flatbed, *RF* ridge and furrow, *BBF* broad bed and furrow, *FYM* farmyard manure, *VC* vermicompost, *DMA* dry matter accumulation, *SEm±* standard error of mean, *LSD* least significant difference.

Not only land configurations but organic nutrient management practices also significantly affected growth and yield attributing characteristics of baby corn (Table 3). All the organic manurial treatments gave significantly higher plant height, LAI, DMA, baby cob length, girth, and weight than control. Among the nutrient management practices, conjoint application of FYM 6 t ha^−1^ + VC 2 t ha^−1^ produced significantly taller baby corn plants (147.67 cm), higher DMA (66.44 g plant^−1^), cob length (7.72 cm), cob girth (5.61 cm) and weight (7.64 g) than control. However, the LAI did not vary significantly between the conjoint application of FYM 6 t ha^−1^ + VC 2 t ha^−1^ and sole application of VC 4 t ha^−1^.

### Productivity and profitability

The average productivity and profitability of baby corn significantly varied with alteration in land configuration (Table 4). The RF and BBF method of sowing had 10.3% and 39.0% higher fresh baby corn yield over the FB (1.36 t ha^−1^), respectively. Substantial improvement in the green fodder productivity of baby corn was also noticed due to land configuration. Both the land configurations i.e., RF (21.67 t ha^−1^) and BBF (22.18 t ha^−1^) produced a significantly higher fresh fodder yield of baby corn over the FB (18.64 t ha^−1^). However, the BBF recorded the maximum enhancement (19.0%) in fresh fodder yield over FB. Baby corn grown on BBF gave the highest gross return (US$1646.5 ha^−1^), net return (US$ 906.1 ha^−1^), and B:C ratio (2.21) followed by RF and the lowest net return (US$ 567.6 ha^−1^) was obtained under FB (Table 4). The BBF sowing generated 35.0, 59.6, and 20.1% higher gross return, the net return, and B:C ratio over the FB, respectively.

**Table 4:** Effect of land configuration and organic nutrient management practices on productivity and economics of baby corn (Five years avg.).

Treatments	Fresh baby corn yield (t ha^−1^)	Fodder yield (t ha^−1^)	Gross returns (US$ ha^−1^)	Net returns (US$ ha^−1^)	B:C ratio
**Land configuration**					
FB	1.36	18.64	1219.7	567.6	1.84
RF	1.50	21.67	1359.0	647.8	1.90
BBF	1.89	22.18	1646.5	906.1	2.21
SEm ±	0.018	0.33	14.3	13.7	0.021
LSD (*p* = 0.05)	0.052	0.97	42.0	40.1	0.062
**Organic nutrients management practice**					
Un-amended control	0.90	13.02	819.2	369.4	1.81
FYM12 t ha^−1^	1.56	18.74	1360.3	699.2	2.05
VC 4 t ha^−1^	1.91	25.56	1707.4	824.3	1.93
FYM 6 t ha^−1^ + VC 2 t ha^−1^	1.96	26.00	1746.9	935.8	2.15
SEm ±	0.021	0.38	16.5	15.8	0.024
LSD (*p* = 0.05)	0.061	1.12	48.5	46.3	0.072

*FB* flatbed, *RF* ridge and furrow, *BBF* broad bed and furrow, *FYM* farmyard manure, *VC* vermicompost, *B:C ratio* benefit to cost ratio, *SEm*± standard error of mean, *LSD* least significant difference.

Among the organic nutrient management practices, the combined application of FYM 6 t ha^−1^ + VC 2 t ha^−1^ produced the highest fresh cobs yield (1.96 t ha^−1^) and fodder yield (26.0 t ha^−1^). However, this was statistically comparable with the sole application of VC 4 t ha^−1^ but remained significantly superior to FYM 12 t ha^−1^ and un-amended control. It was noted that the crop receiving FYM 6 t ha^−1^ + VC 2 t ha^−1^ produced 117.8 and 99.7% higher fresh baby corn and fodder yield, respectively over the plots that did not receive any fertility treatment (control). Application of FYM 6 t ha^−1^ + VC 2 t ha^−1^ registered significantly higher gross (US$ 1746.9 ha^−1^) and net income (US$ 935.8 ha^−1^) over all the organic nutrient management practices, except VC 4 t ha^−1^ which produced a statistically similar gross return. Regarding the B:C ratio, plots receiving FYM 6 t ha^−1^ + VC 2 t ha^−1^ registered significantly higher B:C ratio (2.15) followed by FYM 12 t ha^−1^. The combined application of FYM 6 t ha^−1^ + VC 2 t ha^−1^ recorded 18.8% higher B:C ratio over control.

### Quality

The quality of baby corn cobs was assessed for P, K, Fe, Zn, protein, and ascorbic acid content. Among the land configurations, baby corn cobs produced under BBF treatment had the maximum amount of P (0.64%), K (2.78%), Fe (62.07 ppm), Zn (81.72 ppm), protein (21.63%) and ascorbic acid (93.27 mg 100 g^−1^) followed by the RF. However, cobs produced under FB had the lowest content of P, K, Fe, Zn, protein, and ascorbic acid (Table 5). But, Fe, Zn, and ascorbic acid content in cobs were statistically comparable among the land configurations. The concentration of different minerals (P, K, Fe, and Zn), protein, and ascorbic acid in baby corn cobs was also significantly influenced by various organic nutrient management practices (Table 5). Baby corn cobs produced with organic manures (FYM, VC alone, or in combination) had significantly higher minerals, protein, and ascorbic acid concentration than control. Application of VC 4 t ha^−1^ registered the highest P, K, Fe, and Zn concentration in cobs of baby corn, which was statistically at par with combined application of FYM 6 t ha^−1^ + VC 2 t ha^−1^. Conversely, the application of FYM 6 t ha^−1^ + VC 2 t ha^−1^ registered a significantly higher protein and ascorbic acid content in baby corn cobs. However, the maximum enhancement in protein (35.6%) and ascorbic acid (22.7%) contents in cobs of baby corn over control were recorded under FYM 6 t ha^−1^ + VC 2 t ha^−1^.

**Table 5:** Effect of land configuration and organic nutrient management practices on quality of baby corn (Five years avg.).

Treatments	P (%)	K (%)	Fe (ppm)	Zn (ppm)	Protein content (%)	Ascorbic acid (mg 100 g^−1^)
**Land configuration**						
FB	0.58	2.69	60.17	79.30	18.52	91.98
RF	0.63	2.74	61.72	79.42	19.69	92.27
BBF	0.64	2.78	62.07	81.72	21.63	93.27
SEm ±	0.005	0.02	0.77	0.65	0.26	0.79
LSD (*p* = 0.05)	0.015	0.07	NS	NS	0.77	NS
**Organic nutrient management practices**						
Un-amended control	0.52	2.54	54.25	71.91	16.89	82.82
FYM 12 t ha^−1^	0.61	2.73	61.53	79.11	19.06	87.16
VC 4 t ha^−1^	0.68	2.86	65.14	85.04	20.90	98.41
FYM 6 t ha^−1^ + VC 2 t ha^−1^	0.66	2.81	64.37	84.51	22.91	101.64
SEm ±	0.006	0.03	0.89	0.75	0.30	0.92
LSD (*p* = 0.05)	0.018	0.08	2.61	2.20	0.89	2.60

*FB* flatbed, *RF* ridge and furrow, *BBF* broad bed and furrow, *FYM* farmyard manure, *VC* vermicompost, *P* phosphorus, *K* potassium, *Fe* iron, *Zn* zinc, *SEm±* standard error of mean, *LSD* least significant difference.

### Soil properties

After five cropping cycles, soil pH, ρ_b_, soil organic carbon (SOC) pools, and carbon sequestration rate did not vary significantly among the various land configurations (Table 6). However, the BBF treatment recorded the highest SOC (1.47%), SOC pool (39.4 t ha^−1^), and carbon sequestration rate (0.54 t year^−1^ ha^−1^) followed by the RF. Thus, the organic nutrient management practices significantly influenced the soil pH, SOC, SOC pool, and carbon sequestration rate over control (Table 6). Among the organic nutrient management practices, the application of FYM 12 t ha^−1^ had maximum SOC (1.52%), SOC pool (40.6 t ha^−1^), and carbon sequestration (0.74 t year^−1^ ha^−1^) followed by the conjoint application of FYM 6 t ha^−1^ + VC 2 t ha^−1^. However, after five cropping cycles, the application of VC 4 t ha^−1^ had the maximum increments in soil pH (6.23) over the initial value (6.15). With regards to ρ_b_, all the organic nutrient management practices slightly reduced the soil ρ_b_ (1.33–1.35 Mg m^−3^) compared to the initial value (1.37 Mg m^−3^). However, the application of FYM 12 t ha^−1^ recorded the lowest ρ_b_ (1.33) but it remained at par with VC 4 t ha^−1^ and FYM 6 t ha^−1^ + VC 2 t ha^−1^.

**Table 6:** Effect of land configuration and organic nutrient management practices on soil health after five cropping cycles.

Treatment	pH	Soil organic carbon (%)	Bulk density (Mg m^−3^)	Organic carbon stock (t ha^−1^)	Carbon sequestration (t year^−1^ ha^−1^)
**Land configuration**					
FB	6.18	1.44	1.35	38.9	0.43
RF	6.22	1.46	1.34	39.1	0.48
BBF	6.24	1.47	1.34	39.4	0.54
SEm ±	0.01	0.010	0.02	0.29	0.08
LSD (*p* = 0.05)	NS	NS	NS	NS	NS
**Organic nutrient management practices**					
Un-amended control	6.16	1.37	1.36	37.2	0.11
FYM 12 t ha^−1^	6.20	1.52	1.33	40.6	0.74
VC 4 t ha^−1^	6.26	1.45	1.35	39.1	0.49
FYM 6 t ha^−1^ + VC 2 t ha^−1^	6.23	1.48	1.34	39.8	0.59
SEm ±	0.010	0.011	0.003	0.31	0.05
LSD (*p* = 0.05)	0.028	0.032	0.009	0.89	0.15

*FB* flatbed, *RF* ridge and furrow, *BBF* broad bed and furrow, *FYM* farmyard manure, *VC* vermicompost, *SEm±* standard error of mean, *LSD* least significant difference.

The available N, P, and K in the soil after five cropping cycles moderately improved over the antecedent status. The soil under BBF had the highest available N (386 kg ha^−1^), P (19.0 kg ha^−1^), and K (408.7 kg ha^−1^) followed by RF. The soil under BBF treatment had 6.7, 16.6, and 1.9% higher N, P, and K over FB, respectively (Table 7). Among the organic nutrient management practices, the application of FYM 6 t ha^−1^ + VC 2 t ha^−1^ had the maximum soil available N (390 kg ha^−1^). However, it remained statistically at par with VC 4 t ha^−1^ (382.6 kg ha^−1^) but significantly superior over the rest of the nutrient management practices (Table 6). The maximum available P (19.7 kg ha^−1^) and K (428.5 kg ha^−1^) were observed under VC 4 t ha^−1^ followed by FYM 6 t ha^−1^ + VC 2 t ha^−1^. Nevertheless, soil available P and K were not significantly different between these two treatments.

**Table 7:** Effect of land configurations and organic nutrients management practices on soil health after five cropping cycles.

Treatments	Available nitrogen (kg ha^−1^)	Available phosphorus (kg ha^−1^)	Available potassium (kg ha^−1^)	SMBC (µg g^−1^ soil)	DHA (µg TPFg^−1^ soil h^−1^)	FDA (µg FDA g^−1^ soil h^−1^)	Acid phosphatase (µg p-nitrophenol g^−1^ soil h^−1^)
**Land configuration**							
FB	361.6	16.3	401.1	352.3	17.1	51.9	2.56
RF	374.1	18.5	402.4	364.2	19.8	59.8	3.17
BBF	386.0	19.0	408.7	374.5	21.5	62.3	3.32
SEm ±	2.72	0.27	2.77	1.73	0.23	0.52	0.08
LSD (*p* = *0.05*)	7.98	0.78	NS	5.08	0.69	1.51	0.22
**Organic nutrient management practices**							
Un-amended control	346.7	15.4	374.3	330.3	15.3	44.9	2.65
FYM 12 t ha^−1^	376.4	17.8	397.8	375.8	20.3	56.0	3.09
VC 4 t ha^−1^	382.6	19.7	428.5	371.2	20.2	63.9	3.19
FYM 6 t ha^−1^ + VC 2 t ha^−1^	390.0	18.9	424.6	377.4	22.1	67.1	3.13
SEm ±	3.14	0.31	3.20	2.00	0.27	0.60	0.09
LSD (*p* = *0.05*)	9.21	0.90	9.40	5.87	0.79	1.75	0.26

*FB* flatbed, *RF* ridge and furrow, *BBF* broad bed and furrow, *FYM* farmyard manure, *VC* vermicompost, *SMBC* soil microbial biomass carbon, *DHA* dehydrogenase activity, *FDA* fluorescein diacetate activity, *SEm*± standard error of mean, *LSD* least significant difference.

Land configurations significantly influenced the soil MBC, DHA, FDA, and acid phosphatase activities. The BBF treatment had the highest soil MBC (374.5 µg MBC g^−1^ soil), DHA (21.5 µg TPFg^−1^ soil h^−1^), FDA (62.3 µg FDA g^−1^ soil h^−1^) and acid phosphatase (3.32 µg p-nitrophenol g^−1^ soil h^−1^) followed by the RF (Table 6). However, FB had the lowest soil MBC, DHA, FDA, and acid phosphatase activities. The BBF had 6.3, 25.7, 20.0, and 29.7% higher soil MBC, DHA, FDA, and acid phosphatase over FB, respectively. Among the organic nutrient management practices, the application of FYM 6 t ha^−1^ + VC 2 t ha^−1^ registered the highest soil MBC (377.4 µg MBC g^−1^ soil) but it remained statistically at par with FYM 12 t ha^−1^ (Table 6). With regards to the soil enzymatic reactions, the maximum DHA (22.1 µg TPFg^−1^ soil h^−1^) and FDA (67.1 µg FDA g^−1^ soil h^−1^) activities were noticed in soil under FYM 6 t ha^−1^ + VC 2 t ha^−1^ followed by VC 4 t ha^−1^. However, maximum acid phosphatase activities were observed in soil under VC 4 t ha^−1^. The un-amended control plot had the lowest soil MBC and soil enzymatic activities. The DHA and the FDA had a significant positive correlation with soil pH, SOC, available-P, and soil MBC (Table 8). Conversely, the correlation of acid phosphatase with pH and SOC, and that of SMBC with SOC was not significant (Table 8).

**Table 8:** Pearson correlation analysis among the enzymatic activities viz., DHA, FDA, and acid phosphatase with soil pH, SOC, available-P, and SMBC.

	SOC	AP	pH	SMBC	DHA	FAD	APs
**SOC**							
Pearson correlation	1						
Sig. (2-tailed)							
N	36						
**AP**							
Pearson correlation	0.495**	1					
Sig. (2-tailed)	0.002						
N	36	36					
**pH**							
Pearson correlation	0.361*	0.670**	1				
Sig. (2-tailed)	0.031	0.000					
N	36	36	36				
**SMBC**							
Pearson correlation	0.247	0.570**	0.347*	1			
Sig. (2-tailed)	0.146	0.000	0.038				
N	36	36	36	36			
**DHA**							
Pearson correlation	0.350*	0.715**	0.418*	0.815**	1		
Sig. (2-tailed)	0.037	0.000	0.011	0.000			
N	36	36	36	36	36		
**FAD**							
Pearson correlation	0.549**	0.732**	0.543**	0.731**	0.878**	1	
Sig. (2-tailed)	0.001	0.000	0.001	0.000	0.000		
N	36	36	36	36	36	36	
**APs**							
Pearson correlation	0.020	0.643**	0.247	0.565**	.738**	0.529**	1
Sig. (2-tailed)	0.910	0.000	0.146	0.000	0.000	0.001	
N	36	36	36	36	36	36	36

*SOC* soil organic carbon, *AP* available phosphorus, *SMBC* soil microbial biomass carbon, *DHA* dehydrogenase activity, *FDA* fluorescein diacetate, *APs* acid phosphatase activities.

**Correlation is significant at 0.01 level (2-tailed).

*Correlation is significant at 0.05 level (2-tailed).

## Discussion

Globally, the Eastern Himalayan region of India is known for its high and intense rainfall (> 250 cm annually)^52^. Hence, excess water stress, soil, and nutrient’s losses are the prime challenges for efficient crop production especially during the pre-monsoon and monsoon periods (March to September). Baby corn, a short duration crop, is grown during Kharif /rainy season in the Eastern Himalayan ecosystem, often encounters high and intense rainfall. Hence, safe disposal of accumulated rainwater is a pre-requisite for the successful cultivation of baby corn. It has been well documented that the construction of permanent beds alters the soil topography and substantially reduces the free movement of water^53^. Land configurations like RF and BBF on hilly terrains across the slope facilitate the safe drainage of excess rainwater and reduce the soil and nutrient losses^54,55^.

In the present study, the poor performance of baby corn under the FB was mainly due to water stagnation during the period of active crop growth and development stages which might have retarded the root growth, leaf expansion; dry matter accumulation and photosynthesis rate owing to inadequate oxygen supply in the root zone^56,57^. Furthermore, the crop under the FB system was prone to lodging during heavy rainfall coupled with the intense wind. This might be due to shallow root systems under FB since in saturated soil the roots are confined in the topsoils^30^. Contrary to FB planting, the BBF and RF systems facilitate safe disposal of rainwater, provide good soil aeration, better nutrient availability to baby corn plants leading to the higher LAI, dry matter accumulation, yield attributing parameters, and yield^54,55^. Perhaps, the baby corn grown under the RF and BBF might have a deeper root system^58^ which could tolerate powerful winds during rainy seasons leading to better crop yield over the FB system. Better plant growth and dry matter accumulation under raised bed sowing over flat sowing were also observed in other parts of the world^59,60^. In the present study, RF and BBF systems recorded significantly higher marketable baby corn cobs and green fodder yield over the FB. The BBF recorded 39.0% higher baby corn yield and 19.0% higher green fodder yield over the FB. A 30–50% higher cereal yield with BBF planting system over the FB in high rainfall areas has been reported^61^ previously also. In the present study, beds of 60 cm width and ~ 15 cm height were made under BBF which allowed effective drainage of water from the plant’s root zone and reduced the probability of water-logging and soil compaction by improving the infiltration and rhizospheric aeration^62^ and nutrient availability to plants^57,63^, which ultimately led to better plant growth and yield. Additionally, BBF sowing might have improved the solar radiation interception by crop canopy, thereby reducing the biotic stresses^64^ and increased baby corn cob yield. Several researchers previously reported a higher yield of crops under raised bed sowing over FB in high rainfall areas^33,65,66^. The economic assessment is an important indicator to test the feasibility of any technology. In the present study, the cultivation of baby corn under the BBF system gave significantly higher net return and a B:C ratio over FB sowing. This is ascribed to higher baby corn and fodder yield in the BBF system than others. Higher economic return from crops grown on raised beds in high rainfall areas over FB sowing was also reported by other researchers^54,67^.

Baby corn produced under the BBF and the RF system had higher mineral concentration, protein, and ascorbic acid content as compared to the FB. Both the BBF and the RF facilitate drainage of excess rainwater; reduce water-logging and promote root growth and development which enhances the crop nutrient uptake^30,54^. The lowest concentration of minerals namely P, K, Fe and Zn, protein and ascorbic acid in baby corn produced under FB was attributed to water stagnation which might have reduced the photosynthetic rate^68^, root hydraulic conductivity, nutrient absorption and translocation of photoassimilates^69^ and was perhaps responsible for the poor quality of cobs.

The soil pH, SOC, and ρ_b_ were not significantly affected by the land configuration; however, the BBF system registered a 20.4% higher carbon sequestration rate over the FB. This can be attributed to better root growth and biomass production with a higher quantity of carbon residues that remained in soil after harvesting than others. The BBF improves soil structure and aeration which promotes overall biomass production^30^. Contrary to BBF, the non-stable structure of the soils under the FB developed surface crust that increased soil compaction and reduced root growth thereby leading to poor biomass production. Land configurations significantly influenced the soil available N and P but failed to affect available K in the soil. This may be because the BBF system improves the soil structure and aeration which enhances nutrient mineralization and transformation. The soil MBC, DHA, FDA, and acid phosphates activities also substantially improved in soils under BBF after five cropping cycles. BBF reduced the soil ρ_b_, improved infiltration, and soil pH which helped in improvement in MBC and soil enzymatic activities under high rainfall areas^70^.

The organic nutrient management practices greatly influenced the growth, productivity, profitability, and quality of baby corn cobs. The combined application of FYM and VC had a significant role in maximizing the growth and yield parameters. The combined application of FYM and VC ensured the regular supply of nutrients to the plants particularly N and P which may improve protein synthesis and photosynthesis leading to better plant growth and development than the sole application of either one. The enhancement of plant growth due to the combined application of the FYM and the VC may not only be nutritional but also due to the content of active plant growth-promoting ingredients^71^. It may also be attributed to a balanced supply of nutrients from the combined use of FYM and VC. The combined use of FYM and VC possibly synchronized the demand and supply of nutrients to the baby corn. The combined use of FYM (a wider C: N: P ratio manure) and VC (a narrow C: N: P ratio manure) might have increased the mineralization of native N and mobilization/solubilization of occluded soil P, and also release of growth regulators from VC^72^. Better baby corn growth due to combined use of FYM and VC can be correlated with the effect of VC, which is a rich source of available nutrients^73^ and improvement in soil physico-chemical and biological properties. Hence, the integrated use of FYM and VC has proved as potential organic inputs for yield sustenance of baby corn. Integrated use of FYM + VC gave 2.6% and 25.6% higher baby corn yield over sole application of VC and FYM, respectively. Similarly, the integrated use of FYM + VC was more economical over its sole application. In this study combined use of FYM + VC recorded 13.5% and 33.8% higher net return over VC and FYM-alone, respectively. This may be attributed to the favorable effects of FYM and VC on soil physico-chemical and biological properties^74^ which augment the economic yield. Numerous studies advocated that the nutrients supplying power of organic manure is mainly due to stabilized organic matter content and nutritive elements contained therein^75,76^.

Higher concentrations of minerals in baby corn were directly correlated with the higher concentrations of minerals in manures applied and the soil^76^. Baby corn produced under the VC plots had a higher concentration of P, K, Fe, and Zn but it was statistically comparable with plots receiving the combined application of FYM and VC. Humic substances present in the VC and the FYM have been reported to steadily increase the bioavailability of macro- and micro-nutrients and increase the Zn and Fe content in plant tissues^77^. In this study, the combined application of FYM and VC registered 9.6–20.2% more protein and 3.3–16.6% higher ascorbic acid over the sole application of VC and FYM, respectively, probably due to the better availability of macro and micronutrients to the baby corn plants. The high protein and ascorbic acid content in baby corn under FYM and VC treated plots could also be due to a balanced and consistent supply of nutrition, especially N.

Soil pH and SOC content increased significantly in the plots that received VC or FYM either alone or in combination. In general the application of FYM, 12 t ha^−1^ caused maximum improvement in SOC and C sequestration over other manurial treatments. However, at a given level, the increase in SOC content was more with the combined application of FYM and VC as compared to the sole application of VC. The increase in SOC content may be attributed to the addition of more organic materials through organic manure. Conversely, the maximum improvement in soil pH was noticed under the application of VC. This was due to the slightly higher pH of VC as compared to FYM. Organic manures had low ρ_b_ and high porosity hence, the incorporation of FYM and VC either alone or in combination reduced the soil ρ_b_. Continuous and long term application of FYM along with VC resulted in a 2.2% lower ρ_b_ than control. Long term addition of organic manures substantially reduces the soil ρ_b_ due to buildup of soil organic matter (SOM)^78,79^. Carbon sequestration in soil is mainly governed by the addition of carbon input and carbon stabilization in soil. The combined use of FYM and VC added more stable carbon in the soil, hence, it was found more effective in improving the SOC pool and carbon sequestration rate over VC alone. The beneficial effect of the VC and the FYM on available N, P and K status may be ascribed to the direct addition of these nutrients in an active soil pool. Improvement in the soil available N, P, and K due to conjoint use of organics is also documented under diverse climatic conditions^80–82^. Microbial biomass plays a vital role in regulating the carbon and N transformation in the soil and the amount of microbial biomass is strongly affected by soil and crop management practices. An improvement in the soil's physical properties and C, N, P, and K status and soil microbial biomass due to the addition of organic manure has been reported by several other researchers^83–85^. The addition of organic inputs increases soil MBC and enzymatic activity^86^. Higher soil MBC is an indicator of intensive microbial activities and thus, more putrefaction of SOM^87^. Enhancement in soil enzymatic activities is perhaps a united effect of an increase in microbial biomass and a high degree of enzyme stabilization with humic compounds^88^. Positive effects of long term application of organic manures on soil MBC and soil enzymes were also previously reported^52^. The application of organic manures enhances soil enzymatic activities by increasing SOM and microbial biomass^88,89^. Our results suggest that the alternation in soil enzymatic activities was regulated by types of organic manure. In our experiment, enzymatic activities viz*.,* DHA, FDA, and acid phosphatase were positively correlated with soil pH, SOC, available-P, and SMBC. The significant correlation between soil pH and soil enzyme activities (DHA and FDA) indicated that soil pH could affect the soil enzymatic activities under acidic soils.

## Conclusions

The study proved the hypothesis that broad bed and furrow (BBF) sowing and conjoint application of FYM and VC increased yield and quality of organic baby corn and soil health in the Eastern Himalayan region of India. Data obtained from the present study highlighted the importance of land configuration and integrated organic nutrient management in profitable organic baby corn cultivation in high rainfall hilly areas. Thus, the cultivation of baby corn on BBF produced higher baby corn yield, fodder yield, gross and net return than others. A considerable improvement in the quality of baby corn and soil health was noticed under the BBF whilst compared to that of the FB system. Among the organic nutrient management practices, integrated use of the FYM and the VC was found beneficial in terms of improving growth, productivity, profitability, and quality of baby corn besides soil health. Hence, the cultivation of baby corn on BBF coupled with the integrated application of FYM 6 t ha^−1^ and VC 2 t ha^−1^ may be a viable option for quality production of organic baby corn in the Eastern Himalayas or other similar eco-regions of the world having high and intense rainfall.
